# Artificial Intelligence-Based Recognition of Different Types of Shoulder Implants in X-ray Scans Based on Dense Residual Ensemble-Network for Personalized Medicine

**DOI:** 10.3390/jpm11060482

**Published:** 2021-05-27

**Authors:** Haseeb Sultan, Muhammad Owais, Chanhum Park, Tahir Mahmood, Adnan Haider, Kang Ryoung Park

**Affiliations:** Division of Electronics and Electrical Engineering, Dongguk University, 30 Pildong-ro 1-gil, Jung-gu, Seoul 04620, Korea; haseebsltn@gmail.com (H.S.); malikowais266@gmail.com (M.O.); pipetsupport@naver.com (C.P.); tahirmahmood.cs@gmail.com (T.M.); adnanhaider@dgu.ac.kr (A.H.)

**Keywords:** shoulder arthroplasty, X-ray images, implant classification, deep learning, dense residual ensemble-network, rotational invariant augmentation

## Abstract

Re-operations and revisions are often performed in patients who have undergone total shoulder arthroplasty (TSA) and reverse total shoulder arthroplasty (RTSA). This necessitates an accurate recognition of the implant model and manufacturer to set the correct apparatus and procedure according to the patient’s anatomy as personalized medicine. Owing to unavailability and ambiguity in the medical data of a patient, expert surgeons identify the implants through a visual comparison of X-ray images. False steps cause heedlessness, morbidity, extra monetary weight, and a waste of time. Despite significant advancements in pattern recognition and deep learning in the medical field, extremely limited research has been conducted on classifying shoulder implants. To overcome these problems, we propose a robust deep learning-based framework comprised of an ensemble of convolutional neural networks (CNNs) to classify shoulder implants in X-ray images of different patients. Through our rotational invariant augmentation, the size of the training dataset is increased 36-fold. The modified ResNet and DenseNet are then combined deeply to form a dense residual ensemble-network (DRE-Net). To evaluate DRE-Net, experiments were executed on a 10-fold cross-validation on the openly available shoulder implant X-ray dataset. The experimental results showed that DRE-Net achieved an accuracy, F1-score, precision, and recall of 85.92%, 84.69%, 85.33%, and 84.11%, respectively, which were higher than those of the state-of-the-art methods. Moreover, we confirmed the generalization capability of our network by testing it in an open-world configuration, and the effectiveness of rotational invariant augmentation.

## 1. Introduction

The human shoulder is the most mobile joint of the body. The shoulder may be damaged owing to severe fractures or injuries to the upper arm or severe joint infection. Shoulder surgery is needed when damage to the shoulder joint progresses to such an extent that non-operative procedures cannot resolve the issue or the joint movement causes severe pain. According to the Agency for Healthcare Research and Quality, 53,000 Americans undergo shoulder replacement surgery each year [1]. Total shoulder arthroplasty (TSA) and reverse total shoulder arthroplasty (RTSA) [2] are medical procedures for treating arthritic shoulder joints. With this treatment, a prosthesis is used to repair the damaged joint of the shoulder to re-establish movement and reduce pain. TSA and RTSA are critical for shoulder pain in osteoarthritis. Proper preoperative preparation can help avoid many complications in the revision of TSA and RTSA.

One key surgical step that helps avoid more common complications is identifying prostheses to properly position them. As the morphology of the human shoulder varies from person to person, prostheses are comprised of fixtures and superstructures that can vary by their model, structure, and manufacturer. Therefore, the “one size fits all” idea is not suitable for the treatment of shoulder arthroplasty. Therefore, selecting the correct prostheses model from the right manufacturer for the right patient is very important as personalized medicine. Designing a framework for automatic selection of suitable prostheses for a patient would allow the surgeons to conduct prior and more effective decision-making.

**Figure 1:**
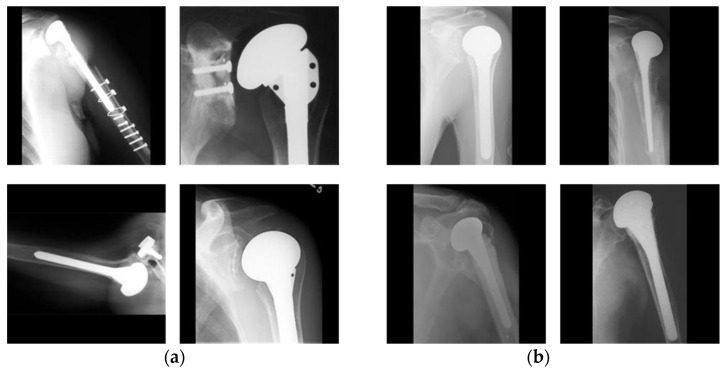
Examples showing high intra-class variabilities and low inter-class variabilities. Examples showing (**a**) high intra-class variability of one manufacturer (Cofield) and (**b**) low inter-class variability. In (**b**), upper-left, upper-right, lower-left, and lower-right images show the cases of four manufacturers of Cofield, Depuy, Tornier, and Zimmer, respectively.

There are many different combinations of device characteristics and surgical approaches, and surgeons often deal with a small number of implants at a time to maximize their expertise with the technology [3]. With a lack of comparable data, surgeons choose which from the few implants they currently offer are appropriate solutions for each patient, rather than choosing from the whole range of alternatives available on the market. However, in some clinical situations, surgeons may believe that only one device is the best option. Older patients, for example, are unlikely to gain additional benefits from a newer implant, but they are at higher risk of surgical problems than younger patients if revision is required [4]. In such cases, selecting a particular implant is crucial. Owing to the limited experience of surgeons with limited implants models, this makes them difficult to work in such situations. Moreover, implants are not identified by medical doctors due to incoherence in documentation and global limitations relating to access to such records, in particular by outside hospital systems [5]. With time, some models of former implants have been halted and their production cut off, whereas new models that differ somewhat from the prior models are being introduced by manufacturers. Moreover, the inclinations of doctors toward certain prostheses change over time. In an investigation carried out by arthroplasty surgeons, 88% of surgeons indicated that implant identification is a critical obstacle to the treatment of an arthroplasty patient [6]. Different prosthesis models require different systems and equipment for replacement and repair, and accurate identification of the model is mandatory. Failure to identify the correct model before surgery results in a waste of healthcare resources, time, and the health of the patient. In some situations, the manufacture and model of the implant might be obscure to surgeons and patients, for example when the original medical procedure is performed outside of the county, and the patients are unable to access their medical records. Over 40% of patients in institutions other than their original arthroplasty are less likely to access outside medical records in a timely manner [5]. As for other reasons why the prosthesis model and manufacturer are unknown, the first original surgery might be performed numerous years before the subsequent surgery, and the patient’s medical information might become lost or unclear. In these cases, medical experts identify a prosthesis through a visual comparison of X-ray images and an implant atlas [7]. This task is tedious, time-consuming, dependent on the surgeon’s experience, and an erroneous recognition can have certain consequences. Therefore, there is a need for an automated method for the identification of prostheses to aid surgeons with pre-operative planning and to save time and medical costs. However, high intra-class variabilities and low inter-class variabilities in shoulder implants appear in X-ray images, as shown in Figure 1, which makes this research extremely challenging.

Despite significant advancements in pattern recognition and deep learning (DL) in the medical field, there has been extremely limited research conducted on classifying shoulder implants. To address these issues, we propose a robust deep-learning-based framework comprising an ensemble of convolutional neural networks (CNNs) to classify shoulder implants in X-ray images. Compared to previous studies, our research is novel in the following five ways.

- To effectively identify shoulder implants, we propose a dense residual ensemble-network (DRE-Net) comprising two CNN models and a shallow concatenation network (SCN). Our network achieves a higher accuracy compared with state-of-the-art studies.
- We propose a rotational invariant augmentation (RIA) to tackle the overfitting problem.
- To check the generalization capability of our network, the proposed DRE-Net is analyzed in different configuration modes of open and closed worlds.
- We analyzed the impact of end-to-end and sequential training of DRE-Net on the testing accuracy of shoulder implant images.
- Our model is publicly available [8] for a fair comparison by other researchers.

The remainder of this paper proceeds as follows. In Section 2, related studies on the classification of different prostheses are described. Section 3 details our proposed classification framework for shoulder implants. In Section 4, the experimental setup and results are presented. Finally, the discussion and conclusions are presented in Section 5 and Section 6, respectively.

## 2. Related Works

Previous studies on implant recognition have classified handcrafted feature-based and deep feature-based methods. Prior to the approach of DL strategies, previous studies have considered handcrafted feature-based methods for implant identification [9,10,11].

DL models have recently contributed pivotal additions in different clinical areas [12,13], including lesion classification [14,15], lesion detection [16,17,18], and lesion segmentation [19,20,21,22]. DL also affects every clinical specialty, including orthopedic surgery [23,24]. Plain film radiographs have been subjected to highly developed DL methods for identification of the elbow, wrist, ankle, and humerus; classification of the hip fracture types and proximal humerus; detection of the presence of arthroplasty and its type; detection of aseptic loosening; and staging the severity of knee osteoarthritis; among other applications [25,26,27,28,29,30,31]. In [32], a DL system was proposed to classify the knee implants of three datasets. The authors used variants of the residual network (ResNet) for different datasets and conducted a classification of two manufacturers and two models. Their network is trained to recognize only two classes, which limits its generalizability. In [33], the authors achieved 99% accuracy by using an artificial intelligence-based DL model to classify knee implants from four manufacturers. In [34], the authors used the visual geometry group (VGG)-16 and VGG-19 models by applying transfer learning to classify dental implants in panoramic X-ray images. Transfer learning with pre-trained networks is effective for learning richer features from large datasets to a small dataset to achieve a high level of accuracy. They manually segmented the panoramic images, and their network was unable to detect the uncropped panoramic image.

In [35], the authors used different CNN models, including SqueezeNet [36], GoogLeNet [37], ResNet-18 [38], MobileNet-v2 [39], and ResNet-50 [38] for the classification of dental implants in X-ray images. They used transfer learning with these pre-trained networks and achieved an accuracy of 90%–97%. In [40], they used a dense convolutional network (DenseNet)-201 [41] CNN with transfer learning to classify three total hip replacement prosthesis models in X-ray images with 100% accuracy. They implemented DenseNet-201 using two different weight initialization methods: (1) a random Gaussian distribution and (2) pre-trained weights of a CNN on the ImageNet database [42]. They demonstrated that a pretrained CNN cannot learn to identify the implant design in X-ray images well. DL also plays a vital role in the detection and classification of bone fractures [27,43]. However, this study was limited to a binary classification of broken and unbroken bones. In [44], a computer-assisted diagnosis (CAD) system based on a hierarchical CNN was designed for the classification of different types of fractures in X-ray images. However, in the case of some classes, the accuracy does not meet the expectations of physicians, and the system still needs to be improved for the classification of subclasses. A deep learning-based study was conducted on the classification of shoulder implants by four manufacturers, where the authors presented comparisons of DL models with different classifiers [45]. Nevertheless, the experiments were only conducted for a closed-world problem. They used the transfer-learning method and did not involve an open-world setting to address real-world problems. In [46], DL was used for the binary classification of shoulder implant models. They used a transfer learning approach and fine-tuned ResNet-18 for binary classification of the existence of arthroplasty implants. Similarly, they used the same approach to distinguish between TSA and RTSA. Finally, they used five fine-tuned models based on ResNet-152 to classify the five TSA models in a binary fashion. However, there is a possibility for an image to be labeled for multiple classes using this method.

To overcome these problems, we propose DRE-Net comprised of two deep CNNs and an SCN to classify shoulder implants in X-ray images. We considered a total of four different classes by manufacturers of 597 unidentified patients related to shoulder implants. We propose a deep feature-based framework for the accurate identification of shoulder implants to ease surgeons. We also address the open-world configuration and found that our model has the capability of generalizability and is therefore applicable to real-world problems.

Table 1 shows comparisons of the strengths and weaknesses of previous studies and our approach for the recognition of implants in X-ray images.

## 3. Proposed Methods

### 3.1. Overview of Proposed Method

**Figure 2:**
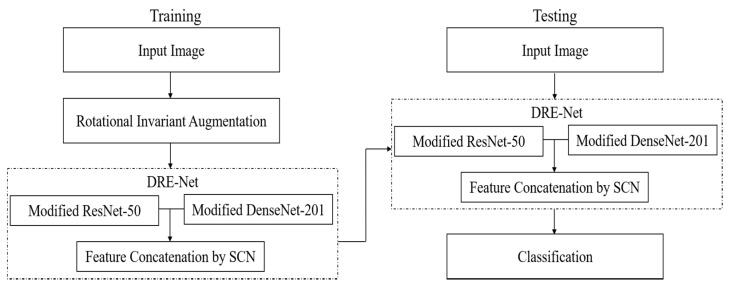
Overall procedure of the proposed method.

Figure 2 shows the overall procedure of our proposed method of shoulder implant classification. During the training phase, input images of 224×224×3 were augmented using the proposed RIA. This technique artificially increases the number of training datasets by the in-plane rotation of each image from 0° to 360° with an interval of 10°. In this way, in addition to the original image, we obtained 36 augmented images from one input. Training is then applied with the proposed DRE-Net, including a modified ResNet-50, a modified DenseNet-201, and an SCN for feature concatenation. During the testing phase, an image is input into the trained DRE-Net, and the final classification of the shoulder implant is conducted based on the output of DRE-Net. Detailed explanations of the proposed RIA and DRE-Net are presented in Section 3.2 and Section 3.3, respectively.

### 3.2. Rotational Invariant Augmentation (RIA)

**Figure 3:**
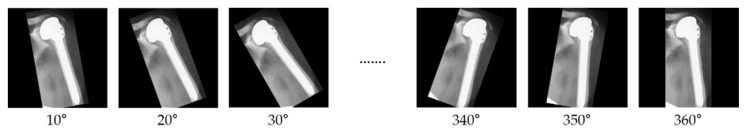
Examples of rotational invariant augmentation (RIA).

The performance of a deep CNN on a dataset, including a small number of images, usually suffers from many different problems, such as an overfitting and a lack of generality. To address this issue, data augmentation has been proposed. Data augmentation includes setting up strategies that upgrade the size and worth of the training dataset with an end goal in which better DL models can be assembled utilizing such strategies [47]. Therefore, we augmented our training dataset based on the in-plane rotation. As a reason for using the in-plane rotation scheme, our dataset consists of implanted shoulder prostheses with rod-like shapes that are easily in-plane rotated in the captured X-ray images, as shown in Figure 1. Data augmentation by an in-plane rotation is applied on each image by rotating the image based on an image center of between 0° and 360°, with an interval of 10°. In this way, we obtained each image with 36 postures at different angles. Figure 3 shows the RIA samples of one image from the Cofield class.

### 3.3. Classification of Shoulder Implants by DRE-Net

**Figure 4:**
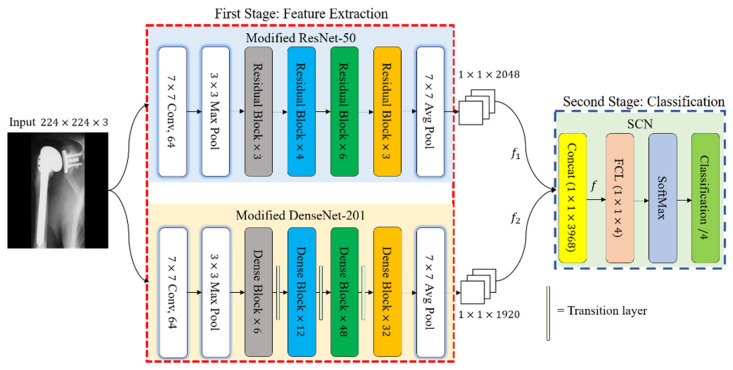
Diagram of our proposed DRE-Net for feature extraction and classification.

In machine learning, ensemble strategies merge various learning algorithms to achieve a preferable performance over any of the constituent models alone [48,49]. In the general frameworks of image classification, the main element is the optimum representation of the visual details or features. Based on this, we propose DRE-Net for the classification of shoulder implants, as shown in Figure 4. In the first stage of DRE-Net, an input image of 224 × 224 × 3 is input to two CNNs of modified ResNet-50 and DenseNet-201, which are modified by removing the fully connected layer (FCL) to extract the optimum features. Explanations of the first stage based on modified ResNet-50 and DenseNet-201 are presented in Section 3.3.1 and Section 3.3.2, respectively. In the second stage of DRE-Net, the SCN obtains two feature vectors (*f*_1_ and *f*_2_ of Figure 4) from the first-stage networks. These features are then concatenated and passed through the FCL and SoftMax layers to classify the shoulder implant into one of the four manufacturers. Detailed explanations of our developed SCN are presented in Section 3.3.3.

#### 3.3.1. Feature Extraction Using Modified ResNet-50

Deep CNNs have demonstrated extreme power in representation learning because they learn the features on a pre-training task and transmit effective knowledge to the target tasks. AlexNet [50], VGG, GoogLeNet, ResNet, and DenseNet are commonly used deep CNNs for transfer learning. The experiments showed that constructing a deep network by copying layers from a learned shallow model leads to a high training error owing to vanishing gradient problems [38]. The residual network has an identity shortcut connection that skips some layers and therefore assists in shielding the network from vanishing gradient issues and improving the performance by deepening the network. Residual nets [38] were first placed in the ImageNet competition [51] for classification, localization, detection, and scoring the first position in common objects in context (COCO) competition for detection and segmentation. In our work, a state-of-the-art deep learning model of ResNet-50 pre-trained on the ImageNet dataset [42] was modified to extract the features for the classification of shoulder implant images.

As shown in Table 2, an image with a resolution of 224×224×3 was given as an input to the first layer labeled “Image Input.” The second layer labeled “Conv 1” was comprised of 64 filters of 7×7×3, which exploits the input image. The convolution layer is a max-pooling layer, which reduced the dimensions of the feature map to a pixel resolution of 56×56×64. Following the max-pooling layer, the layers were grouped into four residual blocks. Each residual block was comprised of two layers of a 1×1 convolution and one layer of a 3×3 convolution. The first group of layers labeled “Conv 2_x” were comprised of three residual blocks, which processed the feature map and down-sampled it to a pixel resolution of 56×56×256. The output feature map of “Conv 2_x” was processed by the second group of layers labeled “Conv 3_x.” This group contained four residual blocks and output a feature map with a pixel resolution of 28×28×512. Similarly, the third group of layers, labeled “Conv 4_x,” contained six residual blocks. It processed the feature map of “Conv 3_x” and generated a feature map with a pixel resolution of 14×14×1024. The last group of layers labeled “Conv 5_x” contained three residual blocks. It processed the feature map of the previous layer and produced a 7×7×2048 sized feature map. Finally, the last average pooling layer named “Average Pooling” was applied with a filter size of 7×7 pixels and obtained a spatial feature vector $f_{1}$ of 1×1×2048. The last three layers of ResNet, labeled “FCL,” “SoftMax,” and “Classification Output” were removed in our modified ResNet to enhance the training convergence and extract only features not considering the classification.

#### 3.3.2. Feature Extraction Using Modified DenseNet-201

With the rapid advancement of CNNs, they are becoming deeper, and the problem of a vanishing gradient has emerged. One solution to this problem is to introduce skip connections between layers, as in the ResNet model. These skip connections guarantee an efficient data stream among the layers in the network. To ensure the stream of maximum information among layers, all layers are associated legitimately with one another, and each layer acquires extra inputs from prior layers and gives its feature map to every single ensuing layer in the DenseNet model [41]. In our work, a state-of-the-art DenseNet-201 pre-trained on the ImageNet dataset [42] was modified to derive the features and classify the shoulder implant images.

As shown in Table 3, an image with a pixel resolution of 224×224×3 was given as an input to the first input layer called an “Image Input.” The second layer, named “Conv 1,” was comprised of 64 filters of 7×7×3, which exploited the input image. Following the convolution layer was a max-pooling layer, which reduced the dimensions of the feature map to 56×56×64 pixels. The layers were then grouped into four dense blocks. Each dense block included a three-sequential composite function with a convolution of 3×3, a rectified linear unit (ReLU) [52], and batch normalization (BN) [53]. The first group of layers, labeled “DenseBlock_1”, which were comprised of six dense blocks, processed the feature map and down-sampled it to a pixel resolution of 28×28×128. The output feature map of “DenseBlock_1” was processed by the second group of layers, labeled “DenseBlock_2.” This group contained 12 dense blocks and output a feature map with a pixel resolution of 14×14×256. Similarly, the third group of layers, labeled “DenseBlock_3,” contained 48 dense blocks and processed the feature map of “DenseBlock_2.” It down-sampled the features, and generated a feature map with a pixel resolution of 7×7×896. The last group of layers, labeled “DenseBlock_4”, contained 32 dense blocks, processed the feature map of the previous layer, and produced a feature map with a pixel resolution of 7×7×1920. Although the architecture contains dense blocks with various filters, the dimensions inside the blocks are equivalent. For compactness of the model and down-sampling of the representations, the transition layer was applied between dense blocks, which comprise the convolution and pooling functions. Finally, the last average pooling layer, named “Average Pooling,” was applied using a filter with a pixel resolution of 7×7, and obtained a spatial feature vector $f_{2}$ with a pixel resolution of 1×1×1920. The last three layers of DenseNet, named “FCL,” “SoftMax,” and “Classification Output” were removed to enhance the training convergence and extract only features not considering the classification. The feature vector $f_{2}$ with 1920 dimensions was concatenated using the 2048-dimension feature vector $f_{1}$ of ResNet-50 in an SCN, and the final classification was made based on the output of the SCN, as shown in Figure 4.

#### 3.3.3. Feature Concatenation and Final Classification by SCN

After extracting the feature vectors from each CNN of the first-stage networks, we further ensembled them to obtain a concatenated feature map using the proposed SCN, as shown in Figure 4. The efficiency of the ensemble learning model was substantially improved. The ensemble model allowed the true objective function to be best approximated within the space of the hypothesis, and the overall performance could be improved using various CNN features [54,55]. We propose an SCN that concatenates two sets of features into a longer feature vector. Table 4 presents the architecture of the SCN. The first layer of the SCN, called “Concat,” takes the inputs from two networks of the first stage with different dimensions and concatenates them. In detail, the feature map $f_{1}$ with pixel dimensions of 1×1×2048 by modified ResNet is concatenated with $f_{2}$ with pixel dimensions 1×1×1920 by modified DenseNet. The Concat layer of the SCN provides a feature map f with a pixel size of 1×1×3968. It then passes through the FCL. The FCL includes a limited number of neurons, taking data from one vector and returning data from another. In general, considering the *j^th^* node of the *i^th^* layer, we can obtain the following equation:

(1) $$z_{i}=\overset{n_{i-1}}{\underset{l=1}{\sum }}\left(w_{j,l}^{\left[i\right]}a_{l}^{\left[i-1\right]}+b_{j}^{\left[i\right]}\right)$$

where in Equation (1), $a^{\left[i-1\right]}$ is the output of the previous layer with dimensions $\left(n_{H}^{\left[i-1\right]}\times n_{W}^{\left[i-1\right]}\times n_{C}^{\left[i-1\right]}\right)$ and is given as input to the FCL by flattening the tensor to a 1D vector with dimensions of $\left(n_{H}^{\left[i-1\right]}\times n_{W}^{\left[i-1\right]}\times n_{C}^{\left[i-1\right]},1\right)$ [56]. The learned parameters at the $l^{th}$ layers are weights $w_{j,l}$ with $n_{l-1}\times n_{l}$ parameters, and bias $b_{j}$ with $n_{l}$ parameters. In addition, $n_{H},\;n_{W},$ and $n_{C}$ represent the height, width, and number of channels, respectively, whereas the final output of the FCL is $z_{i}$. Subsequently, the SoftMax layer is executed. It computes the results of the FCL using the SoftMax function, which compresses the vector z of arbitrary *K* real numbers to a normalized vector of *K* real number probabilities, as a probability distribution ranging between zero and 1 with a probability equivalent to 1 [56]. The SoftMax function is as follows:

(2) $$f{\left(z\right)}_{i}=\frac{e^{z_{i}}}{\sum_{j}^{K}e^{z_{j}}}$$

where in Equation (2), *K* is the number of output classes, and the output $f{\left(z\right)}_{i}$ is the probability for each class. These probabilities are obtained by taking the exponential of each neuron (value) for its class, that is, $e^{z_{i}}$, and dividing by the sum of all exponentials. The denominator part acts as a normalization term to make the sum of all output values equal to 1. Finally, the classification layer computes the final probabilities to determine the class for the image.

### 3.4. Classification Configuration

**Figure 5:**
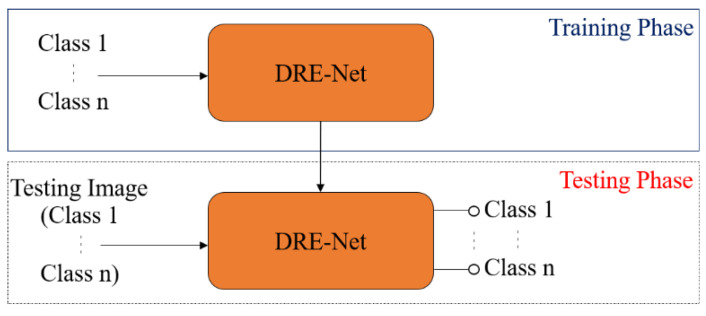
Diagram of closed-world configuration for classification.

In our DRE-Net-based classification of shoulder implants, we designed two configurations of closed-world and open-world configurations. The detailed explanations are as follows: for the closed-world configuration, data from the same class are used for both training and testing. In detail, we applied a 10-fold cross-validation. Therefore, 90% of the data of each class were used for training, and the remaining 10% of the data of the same class were used for testing. This procedure was iterated 10 times, and the average accuracy of 10 trials was obtained as the final classification accuracy. Because the output classes of training and testing were the same, the final classification was made based on the output of DRE-Net, as shown in Figure 5.

**Figure 6:**
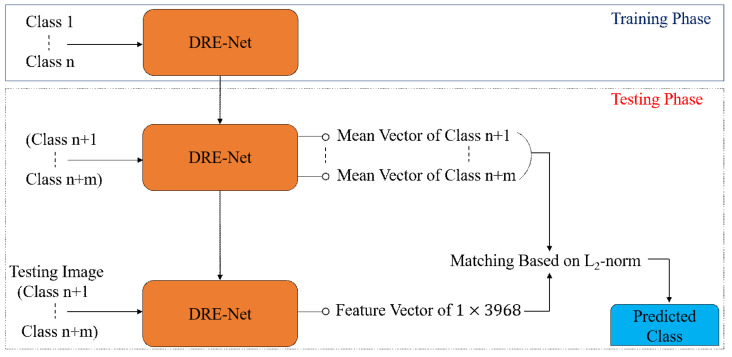
Diagram of open-world configuration for feature extraction and classification.

For the open-world configuration, data from the same class are not used for both training and testing, which means that the classes of training and testing data are completely different, as in general content-based image retrieval systems [57]. We conducted a 2-fold cross-validation considering four output classes. Therefore, the data of classes 1 and 2 were used for training, and the remaining data of classes 3 and 4 were used for testing in the first trial. In the second trial, the training and testing data were exchanged with each other, and the same procedure was repeated. The average accuracy of the two trials was obtained as the final accuracy of classification. Because the output classes of training and testing are different, the final classification cannot be made based on the output of DRE-Net, as in the close-world configuration shown in Figure 5. Instead, the feature vector (1 × 3968) of one testing image is extracted from the first layer (the concatenation layer of Figure 4 and Table 4) of the SCN with trained DRE-Net, and the best matching class is determined based on the L_2_-norm distance (Euclidean distance) between the extracted feature vector and mean vector of the testing classes, as shown in Figure 6. The open-world configuration can reflect the real scenario better than the closed-world configuration, because the data of the untrained class can be obtained in the medical field, as a new manufacturer appears. In this scenario, there is no need to retrain the whole network for all the previous and new classes. Only a reference mean feature vector of the new class (extracted from our network) and its corresponding label (assigned by the medical professional) need to be registered. Then, the model can also work for all the data samples of the new class. In detail, when a new implant model needs to be recognized in a testing phase, the feature vector (1 × 3968) of the image of the new implant model can be extracted from the first layer (the concatenation layer of Figure 4 and Table 4) of the SCN with DRE-Net without additional training. Then, the best matching class can be determined based on the L2-norm distance (Euclidean distance) between the extracted feature vector and the set of reference mean feature vectors.

## 4. Experimental Setups and Results

### 4.1. Dataset and Experimental Setups

**Figure 7:**
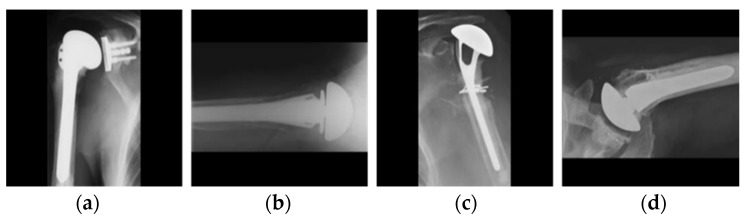
Examples of the dataset: shoulder implants of four different manufacturers: (**a**) Cofield, (**b**) Depuy, (**c**) Tornier, and (**d**) Zimmer.

The dataset used in our research was collected from two different sources comprised of 597 X-ray images of shoulder implant prostheses. This is an open medical dataset that can be used for research purposes. The dataset consists of shoulder prosthesis images of 16 different models from 4 different manufacturers, which were collected from individual manufacturers, surgeons, and the University of Washington [11,45]. One image was captured from each patient in the dataset. The 597 X-ray images of implants are the sum of 83, 294, 71, and 149 of the four manufacturers, Cofield, Depuy, Tornier, and Zimmer, respectively. Figure 7 shows representatives from the dataset, including actual class labels. As shown in Figure 1, the dataset is challenging owing to (1) a high intra-class variance resulting from the various models of the same manufacturer, (2) a small inter-class variance from all X-ray scans of the implants being generally indistinguishable, and (3) a class imbalance. The intra-class variance and class imbalance problems were solved by increasing the dataset size using RIA with sufficient training.

Following the size of the input layer of our model, we resized all images of each class to spatial dimensions with a pixel resolution of 224×224×3 in a portable network graphics (PNG) file format. For the closed-world configuration, we randomly divided the dataset into 10 folds for a cross-validation, as described in Section 3.4. The number of images for the training dataset is not uniform for all classes, and this imbalance problem of the classes degrades the classification performance [58]. To eliminate this issue, we expanded the size of the training dataset by using RIA, but did not perform this augmentation with the testing dataset. Table 5 shows the detailed explanations of the 10-fold cross-validation of the training and testing datasets for the closed-world configuration. C1, C2, C3, and C4 represent the class Cofield, Depuy, Tornier, and Zimmer. We analyzed the performance of state-of-the-art methods using the same experimental protocols. In addition, state-of-the-art methods were also analyzed with online data augmentation and RIA to optimize the results.

A desktop system with the following specifications was used for all experiments in our work: 3.50 GHz Intel^®^ (Santa Clara, CA, USA) Core™ i7–3770K central processing unit [59] with 16 GB RAM, and an NVIDIA (Santa Clara, CA, USA) GeForce GTX 1070 graphics card [60]. A deep learning toolbox with MATLAB R2019b (MathWorks, Inc., Natick, MA, USA) [61] was used on the Windows 10 operating system to implement our RIA algorithm and DRE-Net.

### 4.2. Training of CNN Model

For training DRE-Net, the cross-entropy loss function was used as follows [62]:

(3) $$CE=-\overset{K}{\underset{i}{\sum }}t_{i}log\left(f{\left(z\right)}_{i}\right)$$

where in Equation (3), $f{\left(z\right)}_{i}$ is the probability for each class, which is defined in Equation (2). Cross entropy is simply the negative log of $f{\left(z\right)}_{i}$ for the true label class $t_{i}$. For the true label class, $t_{i}$ becomes 1, whereas it becomes zero for all other classes.

Prior to training the CNNs, all of the dataset images were resized to 224×224×3 pixels. We trained different CNNs involving VGG-16, VGG-19, ResNet-18, ResNet-50, NASNet, DenseNet-201, and our deep DRE-Net for comparison. All CNNs were trained using the stochastic gradient descent (SGD) algorithm [63]. SGD is an optimization method that applies a backpropagation algorithm. The main goal of SGD is to find the optimum parameters for the model based on a mini-batch using the derivative of the loss function. SGD updates parameters, such as the weights and biases for each training instance and label. During the training of the CNN, the loss between the actual label and predicted label is calculated, and the SGD updates the parameters based on the loss function. Owing to the problems of class imbalance and the limited size of the dataset, the dataset was augmented using the proposed RIA. Owing to the small dataset, the filter weights of the first-stage networks of the modified DenseNet and ResNet were initialized using the parameters of pre-trained DenseNet-201 and ResNet-50 along with the ImageNet dataset, respectively. Transfer learning with our training data was then conducted using these CNN models. Transfer learning with pre-trained networks is effective for learning richer features from large datasets to a small dataset to achieve high accuracy. The details of the training parameters for the modified DenseNet, ResNet, and DRE-Net are listed in Table 6. The explanations of these parameters are given in [64]. In our research, we compared the accuracies from sequential training, by which modified DenseNet, ResNet, and SCN were separately trained, and the accuracies from end-to-end training, by which DRE-Net including modified DenseNet, ResNet, and SCN were trained at the same time. The training parameters of the two training cases are presented in Table 6.

**Figure 8:**
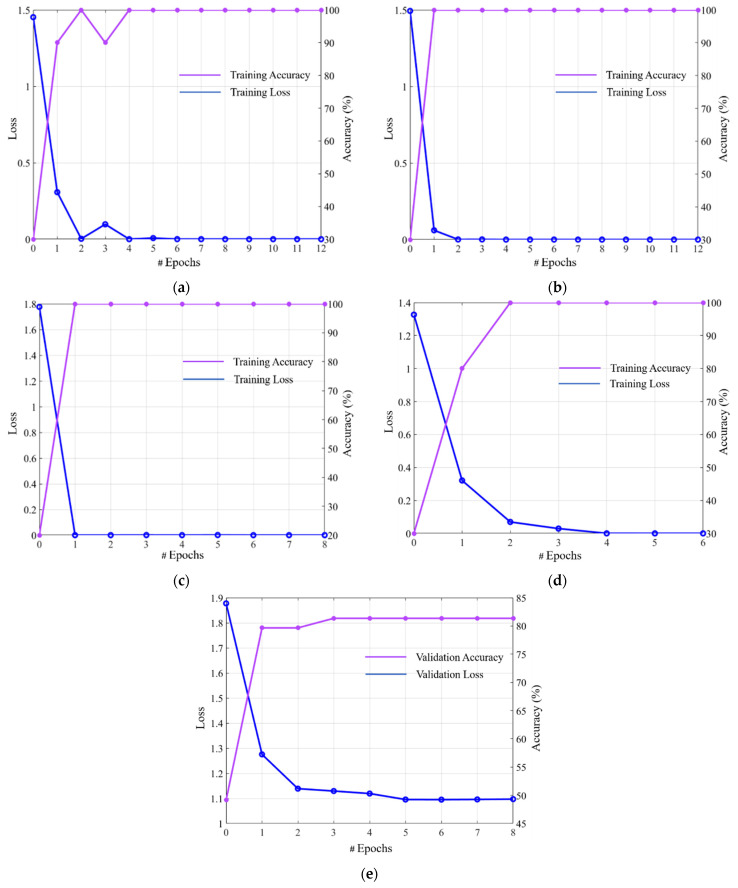
Plots for training losses and training accuracies: sequential training of (**a**) modified DenseNet-201, (**b**) modified ResNet-50, (**c**) SCN, (**d**) DRE-Net (end-to-end training), and (**e**) plots for validation losses and validation accuracies of SCN of (**c**).

The graphs of the training losses and the accuracies through both sequential and end-to-end training are visualized according to the number of epochs, as shown in Figure 8. All networks were converged by increasing the accuracy to 100% while decreasing the loss to 0%, which shows that all networks were successfully trained well. However, the convergence time in terms of loss of the end-to-end training was longer than that of the modified DenseNet, ResNet, and DRE-Net when applying sequential training. In our experiments, we selected 25% of the data as a validation subset and the remaining 75% of the data as a training subset from the training data. We provide the validation losses and accuracies of the proposed SCN (Figure 8c) which shows the better testing accuracies than DRE-Net (end-to-end training) (Figure 8d). Even with the model of training accuracies at 100% (Figure 8c), we could obtain the high validation accuracy and low validation loss as shown in Figure 8e, which confirms the optimal convergence of the proposed network without causing overfitting problem with training data.

### 4.3. Testing and Performance Analysis

We used four qualitative evaluation metrics to assess the performance of our classification network: the accuracy, F1-score, precision, and recall. These metrics are commonly used to evaluate classification frameworks [65] and are calculated as follows:

(4) $$\mathrm{Accuracy}=\frac{1}{K}\sum_{k=1}^{K}\frac{{\mathrm{TP}}_{k}+{\mathrm{TN}}_{k}}{{\mathrm{TP}}_{k}+{\mathrm{TN}}_{k}+{\mathrm{FP}}_{k}+{\mathrm{FN}}_{k}}$$

(5) $$\mathrm{F1-score}=2\times \frac{\mathrm{Precision}\times \mathrm{Recall}}{\mathrm{Precision}+\mathrm{Recall}}$$

(6) $$\mathrm{Precision}=\frac{1}{K}\sum_{k=1}^{K}\frac{{\mathrm{TP}}_{k}}{{\mathrm{TP}}_{k}+{\mathrm{FP}}_{k}}$$

(7) $$\mathrm{Recall}=\frac{1}{K}\sum_{k=1}^{K}\frac{{\mathrm{TP}}_{k}}{{\mathrm{TP}}_{k}+{\mathrm{TN}}_{k}}$$

where K represents the total number of classes, which is equivalent to 4 in our study; $TP_{k}$ is the number of true positives of class *k*, which represents the correctly predicted image from class k; and $FP_{k}$ represents the number of false positives of class *k*, which represents the incorrect prediction of another class into class *k*. In addition, $TN_{k}$ represents the number of true negatives of class *k*, and is the result in which the other class (except for class *k*) is correctly predicted by the model. Finally, $FN_{k}$ represents the number of false negatives of class *k*, which occurs when class *k* is incorrectly predicted into another class using the model.

#### 4.3.1. Ablation Studies

**Figure 9:**
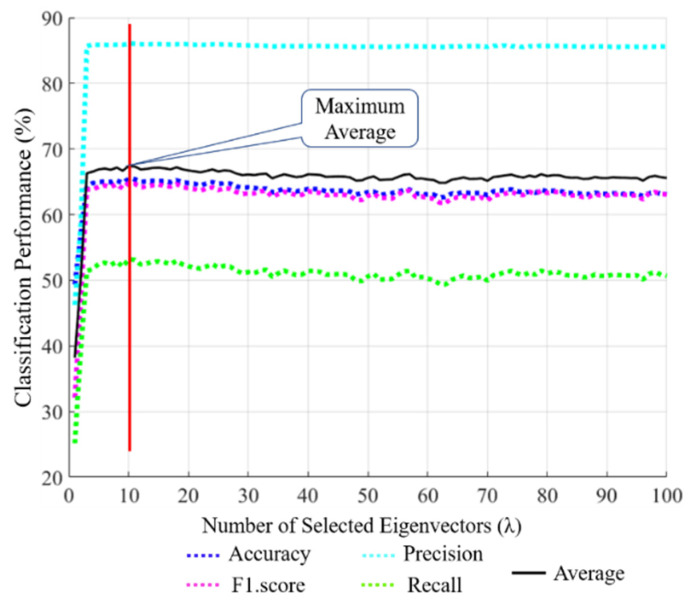
PCA-based performance for different numbers of eigenvectors (λ = 1, 2, 3,…,100).

We studied ablation studies to check the performance and contribution of each component to the overall framework. As the first ablation study, we compared the accuracies of our SCN in Figure 4 with those of the principal component analysis (PCA) + K-NN classifier. A PCA [66] followed by a K-NN [67] was utilized as a post-processing stage to generate the uncorrelated features and scale down the dimensions of the feature vector. The main purpose of applying a PCA is to analyze the discrimination of the selected features (i.e., whether features are distinctive or redundant). From the concatenation layer of a SCN, shown in Figure 4, 1×3968 features are projected into the eigenspace to obtain 3968 eigenvectors and eigenvalues of the training samples. As shown in Figure 9, different eigenvectors are selected to evaluate the PCA for computing the eigenvector (λ), which shows the best performance. As shown in Figure 9, the maximum average performance of λ = 10 was found among all eigenvectors with the training data. Then, the PCA features of the testing samples at λ = 10 were calculated and used as an input to the K-NN classifier. Detailed comparative classification results are shown in Table 7. Although the PCA can reduce the number of dimensions from 1×3968 to 1×10, the classification performance was not higher than that without the PCA-based classification framework (our SCN), as shown in Table 7. This indicates that the high-dimensional features extracted by our deep DRE-Net are already diversified.

Table 8 shows the second ablation study of the shoulder implant classification. As shown in this table, DenseNet-201 and ResNet-50 without the proposed RIA showed lower accuracies by DenseNet-201 and ResNet-50 with RIA. However, the proposed DRE-Net, including DenseNet-201, ResNet-50, and SCN, showed the highest accuracies. The diversity of individually trained ensembles has been reported to be advantageous [68]. Therefore, we compared the results of DRE-Net using sequential and end-to-end training. The results in Table 8 suggest that ensembles of the models benefit from independent training (sequential training). End-to-end training showed a lower performance than sequential training, and the reason for this is that we used high-capacity models, and the ensemble of these models in end-to-end training shows a “model dominance” effect. Table 8 shows that there is a small difference between the results of DRE-Net (end-to-end) and ResNet-50 + RIA compared to those of DenseNet-201 + RIA. That is because DRE-Net (end-to-end) has “model dominance” effect of ResNet-50 + RIA.

**Figure 10:**
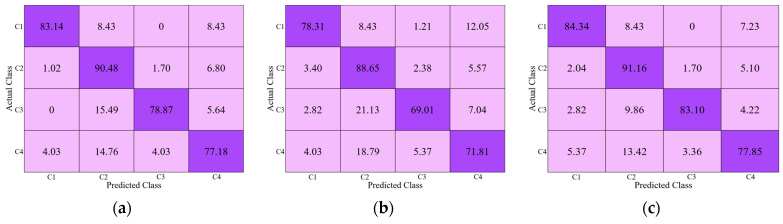
Confusion matrix of (**a**) DenseNet-201 + RIA, (**b**) ResNet-50 + RIA, and (**c**) DRE-Net (sequential training). C1–C4 indicate the classes of four manufacturers of Cofield, Depuy, Tornier, and Zimmer, respectively (unit: %).

Figure 10a–c present the classification performances of the second-best (DenseNet-201 + RIA) and third-best approaches (ResNet-50 + RIA) and our model (DRE-Net (sequential training)) from Table 8 in terms of a confusion matrix. The diagonal values of each table in Figure 10 show the average recall of each class. As shown in Figure 10, our model outperforms both DenseNet-201 + RIA and ResNet-50 + RIA. The reason why class 4 shows lower accuracies by our model than with the other classes is that the data of class 4 have a higher interclass similarity with those of class 2, as explained in Section 5.

#### 4.3.2. Comparison of Proposed DRE-Net with the Subjective Evaluation

**Figure 11:**
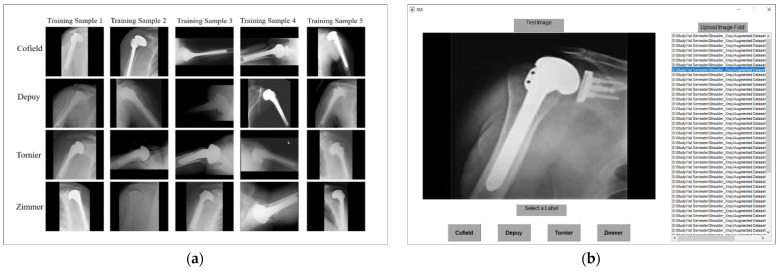
Graphical user interface used for subjective evaluation (**a**) random training samples of each class, which are shown to user during subjective evaluation, (**b**) interface showing all the testing data samples to user one by one for subjective class prediction.

**Figure 12:**
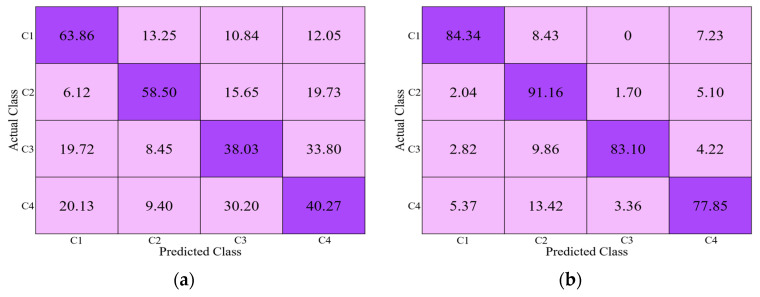
Performance comparison in terms of confusion matrices of (**a**) subjective method and (**b**) the proposed DRE-Net (sequential training). C1–C4 indicate the classes of four manufacturers of Cofield, Depuy, Tornier, and Zimmer, respectively (unit: %).

To highlight the significance of the proposed deep learning method, we additionally performed a subjective evaluation experiment considering the same experimental setup (same testing data samples and 10-fold cross validation). The graphical user interface (GUI) of the experimental protocol was designed in MATLAB R2019b (MathWorks, Inc., Natick, MA, USA) [61], as shown in Figure 11. In detail, a total of 10 individuals (without medical training) participated in this subjective evaluation and visually predicted the class label of all testing data samples one by one for each fold. The demographic details of these participants and subjective performance are given in Table 9. Participants (80% male and 20% female) from three different countries, including 50% from South Korea, 40% from Pakistan, and 10% from Iran took part in this subjective evaluation. All information for experiments was given to participants in advance. Each participant could observe both a set of random training samples of each manufacturer of Figure 11a, and one-fold testing images which is the 10% of the data of Figure 11b at the same time. In this way, each testing-fold samples were provided to each person to perform 10-fold cross validation. The group evaluated all of the testing images of each fold, and assigned the appropriate label to each sample of Figure 11b by visually comparing the training set as shown in Figure 11a. The average time calculated for the evaluation of one participant was about twenty minutes. Once all individuals had completed the evaluation, the average performance of each fold was calculated as shown in Table 9. Finally, we obtained the average performance (as confusion matrix, average accuracy, F1-score, precision, and recall) of this subjective evaluation and compared them with the proposed DRE-Net as presented in Figure 12 and Table 10. It can be observed that our proposed DRE-Net shows the superior performance over subjective evaluation with average performance gains of 33.67%, 35.15%, 36.47%, and 33.83% in terms of accuracy, F1-score, precision, and recall, respectively.

In addition, as shown in Figure 12a, the correct classification accuracy by human subject with Cofield data (C1) was 63.86% which was much lower than that by our proposed method of 84.34%. These results confirm that it is visually difficult to discriminate the data of C1 from Figure 1a, and we can tell that there exist the differences among those intra models.

#### 4.3.3. Comparisons of Proposed DRE-Net with the State-of-The-Art Methods

The performances of various state-of-the-art methods [38,41,45,46,69,70] were compared with those of our approach. Table 11 shows the performance comparisons by the state-of-the-art methods and the proposed method without data augmentation, and ResNet-50 [38] outperformed the other methods. In this case, all methods were compared without a data augmentation for a fair comparison. Table 12 shows the performance comparisons by the state-of-the-art methods and the proposed method with data augmentation (through a random in-plane rotation and translation), which shows higher accuracies than those listed in Table 11. The results in most cases show that ResNet-50 [38] and DenseNet-201 [41] outperformed the other methods. In this case, all methods were compared with the data augmentation (random in-plane rotation and translation) for fair comparisons. However, our proposed model does not produce state-of-the-art results with this augmentation technique, as shown in Table 12. This demonstrates that different augmentation techniques have different impacts on the neural networks.

As can be seen in Table 13, when the performances are compared between the state-of-the-art methods and the proposed method with RIA, a 4.18% performance gain was shown in the average accuracy of DenseNet-201 with ResNet-50. In addition, NASNet exhibited a 1.34% performance decrease in terms of the average accuracy with ResNet-50. Among all methods applied, our approach (DRE-Net (sequential training)) outperforms all other state-of-the-art methods. In this case, all methods were compared with RIA for a fair comparison. In addition, we can confirm that the accuracies of Table 13 are higher than those of Table 11 and Table 12 in most cases. For fair comparison, the weights of the CNN models were pre-trained on the ImageNet dataset, and transfer learning was performed again with our training data in all experiments presented in Table 11, Table 12 and Table 13.

We evaluated the deep models using a 10-fold cross-validation and calculated the mean scores. To verify that the difference between mean scores was statistically significant, a *t*-test [71] was conducted. This test is based on a null hypothesis (H), which states that the performances of our model and the other approaches are not expected to be different (i.e., H = 0). The T-test is carried out to verify the substantial disparity between our model and the second-best [41] and third-best [38] baseline models in Table 13. Our sample size was small and increased the complexity of the statistical analysis. In detail, as the sample size decreases, the chance that every measured mean value is the same as the real total mean value decreases and the degree of uncertainty about the true value of the mean increases. Therefore, we conducted a *t*-test by combining 10-fold cross-validation values of the accuracy, F1-score, precision, and recall. The null hypothesis is rejected when there is less than a 5% chance of validity. The results in Table 14 show that the *p*-values calculated by the second- and third-best methods with our model are 0.03 (<0.05) and 7.84 × 10^−9^ (<0.001%), respectively, which demonstrates the effective distinction between our model and the other approaches. The *p*-value (0.03) for the second-best model shows that the null hypothesis is rejected at a 97% confidence level and shows a significant difference between our approach and the second-best model. In the case of the third-best model, the *p*-value (7.84 × 10^−9^) indicates a significant difference between our approach and the third-best model, and the null hypothesis is rejected at a 99% confidence level.

## 5. Discussions

In this study, we implemented two spatial feature extraction networks using a densely connected convolution network and a residual neural network. In the first stage, our proposed model envisages the spatially extracted features of both networks, which eventually leads to better results compared to other state-of-the-art classification networks. In the second stage, the proposed SCN further processes the spatial features, and therefore, ideal spatial features are extracted to achieve the best result. The architecture of the modified DenseNet model is shown in Table 3, and shows various dense blocks and transition layers used to exploit the optimal spatial features of the input image and achieve superior outcomes over other CNN models.

**Figure 13:**
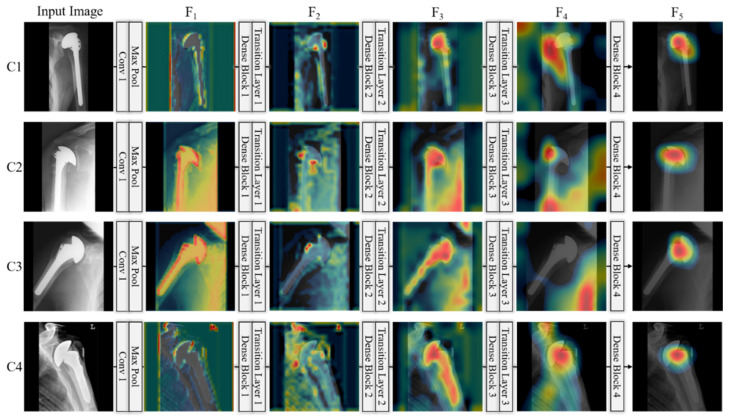
Class activation maps for given inputs of four classes (C1–C4), which are extracted from modified DenseNet-201 of Table 3. C1–C4 indicate the classes of four manufacturers, Cofield, Depuy, Tornier, and Zimmer, respectively.

**Figure 14:**
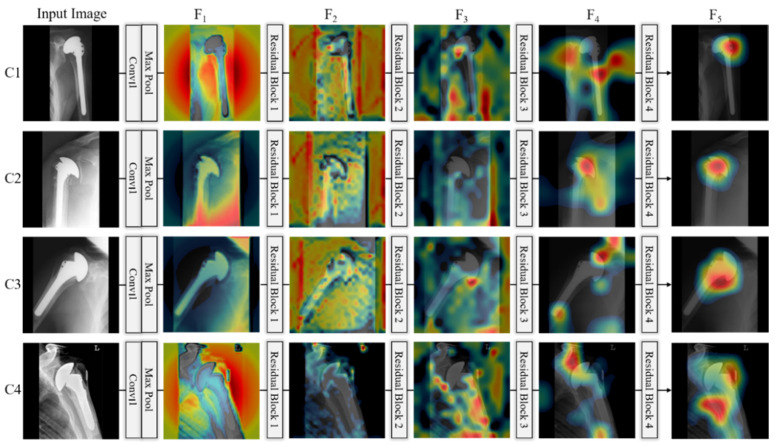
Class activation maps for given inputs of four classes (C1–C4), which are extracted from modified ResNet-50 of Table 2. C1–C4 indicate the classes of the four manufacturers, Cofield, Depuy, Tornier, and Zimmer, respectively.

In this section, we generate class activation maps to illustrate the performance of the achievements of the modified DenseNet. Figure 13 shows the discriminative image regions used by the modified DenseNet to identify the class. The activation maps calculated for each dense block are represented using a pseudo color scheme [72]. The left column in Figure 13 shows the input images of four classes (C1–C4) given to DenseNet to learn its features, and it can be seen that the activation maps (F_1_, F_2_, …, F_5_) become salient after processing through each dense block. Finally, we can obtain class-specific regions (activation map F_5_) that provide the specific visual pattern for each class, which ensures that DenseNet learns the features well. Similarly, we generated class activation maps to illustrate the performance of the modified ResNet. The architecture of the modified ResNet model is listed in Table 2, and shows various residual blocks used to exploit the optimal spatial features of the input image. Figure 14 shows the discriminative image regions used by the modified ResNet to identify the class. The left column in Figure 14 shows the input images of four classes (C1–C4) given to ResNet to learn its features and activation maps calculated by each residual block, which are represented by a pseudo color scheme [72]. The activation maps (F_1_, F_2_, …, F_5_) become prominent after processing through the residual blocks. Ultimately, we can obtain class-specific regions (activation map F_5_) that provide a specific visual pattern for each class. However, as shown in Figure 14, the activation map for class 4 (Zimmer) does not clearly match visually distinct patterns. For a fair comparison between first-stage networks, we used the same input images of different classes to generate activation maps in Figure 13 and Figure 14. The activation maps for class 4 generated by DenseNet and ResNet are quite different. The activation map generated by DenseNet for class 4 is the representation of its visually discriminated region, as shown in the last row of Figure 13, whereas that generated by ResNet for the same class shows a deviation from the discriminated region, as shown in the last row of Figure 14. This indicates that ResNet made predictions not on the head of the implants, which is a discriminated part, but on the background. Therefore, ResNet does not make a decision well for class 4 to learn the features. Moreover, as shown in Figure 10, the confusion matrix of the first-stage networks shows that ResNet has 5.37% less average recall than DenseNet for class 4. In addition, the activation map generated by ResNet-50 for class 3, as shown in the third row of Figure 14, is not focused on the head and is larger than that generated by DenseNet-201, shown in the third row of Figure 13. Therefore, the recall of ResNet-50 is much lower than that of DenseNet-201, as indicated in Figure 10. A similar analysis can be made for class 1. The activation map generated by ResNet-50 for class 1, as shown in the first row of Figure 14, does not accurately exist in the head area compared to that by DenseNet-201, as shown in the first row of Figure 13. Therefore, the recall of ResNet-50 is lower than that of DenseNet-201, as shown in Figure 10.

Finally, the final class activation maps (F_5_) of the first-stage networks are processed by the proposed SCN for final classification after passing through their respective average pooling layers. A class activation map for the second-stage network cannot be generated. The reason for this is that, in the second stage network, the feature vector is 1 × 1 × 4, and it lacks the visual information. Moreover, the ability of visual object detection by convolution layer was lost when FCL was used for classification in the second stage network. The fundamental difference between the SCN and first-stage networks is the processing of the feature maps. DenseNet and ResNet extract and process the feature maps of an image independently, whereas SCN combines the connectivity of both networks and processes their feature maps. In this way, an optimal representation of the spatial features is generated, which ultimately leads to a better performance in the classification of various types of shoulder prostheses.

We also computed the performance of our proposed network for an open-world configuration. For the open-world configuration, we conducted two-fold experiments by splitting the datasets into two halves, as explained in Section 3.4. The first half was used for training, while the other half was used for testing. Similar to the closed-world configuration, the training dataset in the open-world configuration is augmented using RIA. The main step in the open-world setup is to judge the real class label of the query image by calculating its similarity score with the class mean features. Thus, the Euclidean distance can be used to predict a class label for the query image. Owing to the limited number of classes (i.e., 4), we used two-fold cross-validation. Table 15 displays the details of the two-fold cross-validation of the training and testing datasets for the open-world configuration. Table 16 shows the experimental results of our proposed model, and the second- and third-best models are shown in Table 13 for the open-world configuration. There is a 0.72% performance gain in the average accuracy of our model over the second-best model and 2.4% over the third-best model.

**Figure 15:**
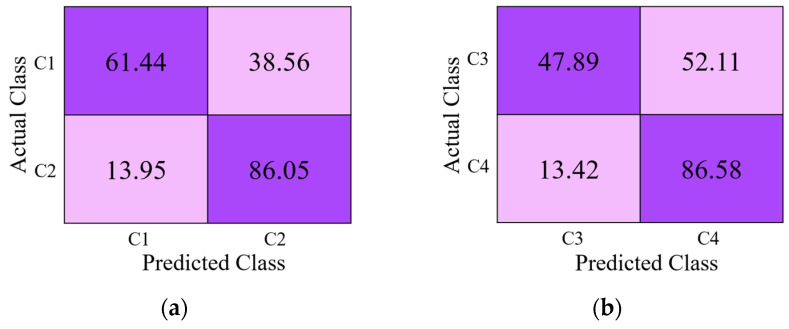

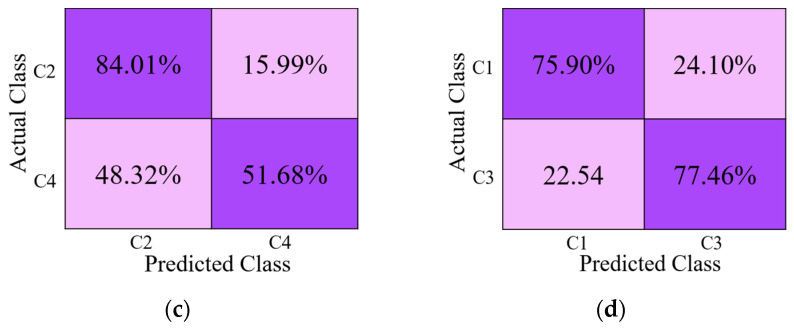
Performance of the proposed network considering open-world setting as confusion matrices (**a**) 1st fold-A (using C1 and C2 in testing), (**b**) 1st fold-B (using C3 and C4 in testing), (**c**) 2nd fold-A (using C2 and C4 in testing), and (**d**) 2nd fold-B (using C1 and C3 in testing).

In this section, we also measured the performance of the proposed network in terms of confusion matrices considering open-world setting, as shown in Figure 15. In the 1st fold-A and -B, Tornier (C3), Zimmer (C4) (Figure 15b) and Cofield (C1), Depuy (C2) (Figure 15a) are used in testing, respectively. Similarly, in the 2nd fold-A and -B, Depuy (C2), Zimmer (C4) (Figure 15c) and Cofield (C1), Tornier (C3) (Figure 15d) are used in testing, respectively. As shown in these figures, the average value of correct classification ((84.01 + 51.68)/2(%)) with the testing of C2 and C4 (Figure 15c) is lower than those with the testing of C1 and C2 (Figure 15a) and C1 and C3 (Figure 15d). However, it is higher than that with the testing of C3 and C4 (Figure 15b). These results mean that the similarity between C2 and C4 does not give much effect on testing by open-world configuration compared to that by closed-world configuration. That is because the number of classes in the testing of open-world configuration (two classes) is half of that of closed-world configuration (four classes), which increases the inter-distance between two classes and consequently reduces the effect of similarity of C2 and C4 on testing accuracy of open-world configuration. In the open-world configuration mode, which is more complicated and challenging than the closed-world configuration mode, our model performs the best and is likely applicable to real-world problems as well.

**Figure 16:**
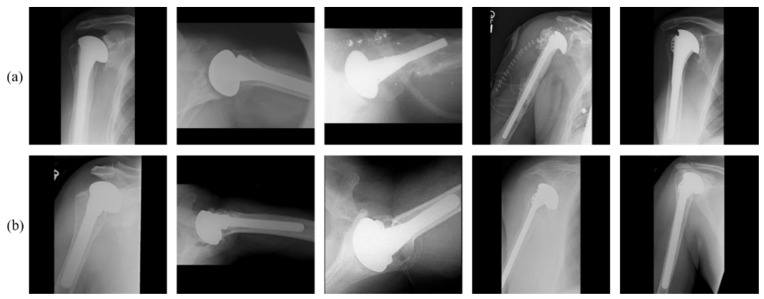
The high inter-class similarity between the two classes: (**a**) class 2 (Depuy) and (**b**) class 4 (Zimmer).

We analyzed the false-positive and false-negative cases of our classifier and found that the reasons for the erroneous classification are structural similarities of the prostheses and the limited size of the dataset. For example, in Figure 10, the confusion matrix of our proposed model shows a lower average recall of class 4 (Zimmer) than that of the other classes. This is because the size (the number of images) of class 4 is two times less than that of class 2 (Depuy) with a high inter-class similarity between them, as can be seen in Figure 16. However, we maintain the sizes of the classes using RIA, although the class imbalance problem remains. It should be considered that the class imbalance problem is still an open issue [73], and thus various solutions are not guaranteed to be optimal. In addition, we analyzed the two-fold experiments for the open-world configuration owing to the limited number of classes. We plan to increase the number of folds in the future by increasing the number of classes. We trained two separate CNNs to extract the features and ensemble them using an SCN. This approach increases the training time owing to the large number of parameters required but makes the model more robust.

## 6. Conclusions

In this study, we proposed the use of DRE-Net by combining features for shoulder implant classification in X-ray images based on two independent models: modified ResNet-50 and DenseNet-201. This framework automatically detects the prostheses by the manufacturer and aids the surgeons to fit it in the patient’s body by their anatomy as personalized medicine. We analyzed the application of different deep learning models for the classification of shoulder implants by the manufacturer, and compared them with the ensemble of two deep learning models. The ensemble of models using the proposed SCN minimizes the weaknesses of each individually and takes advantage of the strengths of both. To further improve the efficiency of the classification, we proposed the application of RIA and increased the results by 8.87%. We discovered that independent (sequential) training of ensemble models shows better performance than end-to-end training. Although the dataset is relatively small, we obtained the optimum results for shoulder implant classification by integrating transfer learning, ensemble learning, feature concatenation, and RIA. We also examined our model for an open-world configuration and achieved the best results compared to the other deep models, which demonstrates the generalizability of our approach. As reported in previous research [11,45,46], the usage of computer-based algorithms can do better to identify shoulder arthroplasty implants compared to medical experts, which can reduce the risk of delayed operations, perioperative morbidity, and overuse of resources due to lack of correct identification of shoulder arthroplasty implants. Based on these motivations, previous research [11,45,46] has also studied the computer-based algorithms for the identification of shoulder implants. This study is helpful for personalized shoulder arthroscopy and researchers working on X-ray image-based implant recognition.

In the future, we plan to upgrade the results and reduce the training time of the proposed technique by establishing a custom-built model. We also plan to extend this work by adding additional manufacturers and classifying shoulder implants using the models. In addition, the class imbalance problem and increased number of classes for open-world configurations will also researched in the future.

## Figures and Tables

**Table 1:** Comparisons between our proposed and previous methods for implant recognition in X-ray images.

Category	Type	Methods	# Classes	Results	Strength	Weakness
Handcrafted feature-based	Knee	Template matching [9]	1	70% to 90% accuracies	- Uses a simple image processing technique including Sobel operator, binarization, and template matching - Computationally efficient	Requires 3D CAD models for template generation of implants
	Dental	Active contours + K-nearest neighborhood (K-NN) [10]	11	91% of the known implants are recognized	- Optimal initial location of the contour can be selected by their method - Uses simple machine learning algorithm for classification	- K-NN classifier is time-consuming for large numbers of features - Because of the large number of dental implant models, their approach returns a set of possible candidate results for identifying new implants and needs a user interaction to verify the candidate result
	Shoulder	Hough transform + histogram equalization + mean shift filter [11]	4	77% precision, and 64% F-measure	- Uses conventional image processing schemes involving bilateral filter, mean shift filter, and a median blur filter - Develops a pre-processing tool for training a classifier	Segmentation performance is dependent on the growing approach of seed region
Deep feature-based	Knee	Pre-trained CNN [32]	2	100% sensitivity, and 100% specificity	- High classification performance - Precisely determines the presence of total knee arthroplasty (TKA) - Accurately classifies the TKA and unicompartmental knee arthroplasty (UKA)	Classification is performed in a binary fashion (the presence of implant)
		Pre-trained CNN [33]	9	99% accuracy, 95% sensitivity, and 99% specificity	High classification performance	Pre-processing is needed and computationally expensive
	Dental	Pre-trained CNN [34]	11	93.5% accuracy, 91.6% F-measures	High average classification accuracy with a small dataset of panoramas	VGG network can be replaced with the state-of-the-art networks
		Pre-trained CNN [35]	4	96% to 97% accuracies	High classification performance and computationally efficient	Their method is unable to detect several implants simultaneously
	Hip	Pre-trained CNN [40]	3	100% accuracy	High classification performance	- Requires high processing power for extensive training - Uses only one post-surgery anteroposterior (AP) X-ray per patient
	Shoulder	Pre-trained CNN [46]	2	95% sensitivity, and 90% specificity to classify TSA and RTSA	- High accuracy to detect the existence of shoulder arthroplasty - High sensitivity to classify TSA and RTSA	Classification is performed in a binary fashion (the presence of implant)
		Pre-trained CNN [45]	4	80.4% accuracy, 80% precision, 75% recall, and 76% F1-score	- First deep learning based-approach to classify the manufacturers of shoulder implants - Higher classification accuracy than non-deep learning-based methods	- Accuracies are needed to be enhanced - Performance was measured only by closed-world configuration
		DRE-Net (Proposed)	4	85.92% accuracy, 84.69% F1-score, 85.33% precision, and 84.11% recall	- High classification accuracy - Applicable to real-world problems by considering both closed-world and open-world configurations	Requires more training time

**Table 2:** Layer configuration details of modified ResNet-50.

Layers Name	Output Feature Map Size	Kernel Size	Number of Iterations
Image Input	224×224×3	-	-
Conv 1	112×112×64	7×7 conv	1
Max Pooling	56×56×64	3×3 max pool	1
Conv 2_x	56×56×256	1×1 conv 3×3 conv 1×1 conv	3
Conv 3_x	28×28×512	1×1 conv 3×3 conv 1×1 conv	4
Conv 4_x	14×14×1024	1×1 conv 3×3 conv 1×1 conv	6
Conv 5_x	7×7×2048	1×1 conv 3×3 conv 1×1 conv	3
Average Pooling	1×1×2048	7×7 avg pool	1

**Table 3:** Layer configuration details of modified DenseNet-201.

Layer Name	Output Feature Map Size	Kernel Size	Number of Iterations
Image Input	224×224×3	-	-
Conv 1	112×112×64	7×7 conv	1
Max Pooling	56×56×64	3×3 max pool	1
DenseBlock_1	56×56×256	$\begin{array}{c} 1\times 1\;conv\\ 3\times 3\;conv \end{array}$	6
Transition Layer	28×28×128	$\begin{array}{c} 1\times 1\;conv\;\\ 2\times 2\;\;avg\;pool \end{array}$	1
DenseBlock_2	28×28×512	$\begin{array}{c} 1\times 1\;conv\\ 3\times 3\;conv \end{array}$	12
Transition Layer	14×14×256	$\begin{array}{c} 1\times 1\;conv\\ 2\times 2\;avg\;pool \end{array}$	1
DenseBlock_3	14×14×1792	$\begin{array}{c} 1\times 1\;conv\\ 3\times 3\;conv \end{array}$	48
Transition Layer	7×7×896	$\begin{array}{c} 1\times 1\;conv\\ 2\times 2\;avg\;pool \end{array}$	1
DenseBlock_4	7×7×1920	$\begin{array}{c} 1\times 1\;conv\\ 3\times 3\;conv \end{array}$	32
Average Pooling	1×1×1920	7×7 avg pool	1

**Table 4:** Layer configuration details of SCN.

Layers Name	Output Feature Map Size	Kernel Size	Number of Iterations
Concat	1×1×3968	-	1
Fully Connected	1×1×4	-	1
SoftMax	1×1×4	-	1
Classification	4	-	1

**Table 5:** Summary of 10-fold cross-validation of training and testing data for closed-world configuration (unit: images).

Validation	Training		Testing				Total
	Original	Augmented	C1	C2	C3	C4	
1st fold	538	19,368	8	29	7	15	19,965
2nd fold	536	19,296	9	30	7	15	19,893
3rd fold	538	19,368	8	29	7	15	19,965
4th fold	537	19,332	8	30	7	15	19,929
5th fold	536	19,296	9	29	8	15	19,893
6th fold	539	19,404	8	29	7	14	20,001
7th fold	537	19,332	8	30	7	15	19,929
8th fold	538	19,368	8	29	7	15	19,965
9th fold	536	19,296	9	30	7	15	19,893
10th fold	538	19,368	8	29	7	15	19,965

**Table 6:** Parameters for network training.

Methods		Number of Epochs	Mini-Batch Size	Learning Rate	Momentum Term	L2-Regularization	Learning Rate Drop Factor
Sequential training	Modified DenseNet-201	13	10	0.001	0.9	0.0001	0.1
	Modified ResNet-50	13	10	0.001	0.9	0.0001	0.1
	SCN	9	10	0.001	0.9	0.0001	0.1
End-to-end training	DRE-Net	7	10	0.001	0.9	0.0001	0.1

**Table 7:** Performance comparisons of our proposed SCN using a PCA and a K-NN (unit: %).

Fold	Performance without a PCA (our SCN)				Performance with PCA (λ = 10) + K-NN			
	Accuracy	F1-Score	Recall	Precision	Accuracy	F1-Score	Recall	Precision
10-Fold Average	85.92	84.69	84.11	85.33	57.94	48.04	40.60	60.17

**Table 8:** Performance comparisons of each sub-network and proposed DRE-Net by end-to-end or sequential training (unit: %).

Methods	Accuracy	F1-Score	Precision	Recall
ResNet-50 [38]	66.70	62.02	64.67	59.83
DenseNet-201 [41]	55.76	47.55	49.73	45.73
ResNet-50 + RIA	80.57	78.02	79.21	76.95
DenseNet-201 + RIA	84.75	83.76	85.21	82.42
DRE-Net (end-to-end)	81.55	79.12	80.77	77.66
DRE-Net (sequential)	85.92	84.69	85.33	84.11

**Table 9:** Demographic details of different subjects and their subjective evaluation results.

Demographic Details				Subjective Performance (%)			
Participant Index	Age	Nationality	Sex	Accuracy	F1-Score	Precision	Recall
1	28	Pakistan	Male	57.63	53.40	53.51	53.29
2	28	Pakistan	Male	55.74	48.86	49.23	48.49
3	23	South Korea	Male	50.85	55.35	55.51	55.19
4	32	Pakistan	Male	48.33	48.43	46.90	50.06
5	27	South Korea	Male	50.82	45.58	45.51	45.66
6	29	South Korea	Male	55.17	45.67	45.13	46.23
7	42	Iran	Female	58.33	54.87	52.92	56.96
8	27	South Korea	Female	45.76	42.83	41.84	43.88
9	32	Pakistan	Male	52.46	46.77	46.63	46.90
10	28	South Korea	Male	47.46	53.68	51.47	56.09

**Table 10:** Average performance comparison (10-folds) between subjective evaluation and the proposed DRE-Net (unit: %).

Methods	Accuracy	F1-Score	Precision	Recall
Subjective Method	52.25	49.54	48.86	50.28
DRE-Net (sequential)	**85.92**	**84.69**	**85.33**	**84.11**

**Table 11:** Performance comparisons of state-of-the-art methods and the proposed approach without data augmentation. Averages from a 10-fold cross-validation are shown (unit: %).

Methods	Accuracy	F1-Score	Precision	Recall
VGG-16 [45,69]	58.70	45	54	45
VGG-19 [45,69]	63.60	54	61	53
ResNet-18 [38,46]	66.13	60.86	64.25	58.13
ResNet-50 [38]	**66.70**	**62.02**	**64.67**	**59.83**
NASNet [45,70]	64.50	54	62	52
DenseNet-201 [41]	55.76	47.55	49.73	45.73
Proposed	58.10	50.82	51.78	49.96

**Table 12:** Performance comparisons of the state-of-the-art methods and proposed approach with data augmentation by random in-plane rotation and translation. Averages from a 10-fold cross-validation are shown (unit: %).

Methods	Accuracy	F1-Score	Precision	Recall
VGG-16 [45,69]	74	69	72	68
VGG-19 [45,69]	76.20	70	75	69
ResNet-18 [38,46]	70.82	65.93	68.02	64.38
ResNet-50 [38]	80.56	**77.66**	79.49	76.02
NASNet [45,70]	80.40	76	**80**	75
DenseNet-201 [41]	**80.57**	77.60	79.05	**76.32**
Proposed	77.05	74.80	76.93	73.07

**Table 13:** Performance comparisons of the state-of-the-art methods and proposed approach with RIA. Averages from a 10-fold cross-validation are shown (unit: %).

Methods	Accuracy	F1-Score	Precision	Recall
VGG-16 [45,69]	68.85	66.90	66.82	67.22
VGG-19 [45,69]	66.54	63.54	63.81	63.35
ResNet-18 [38,46]	77.41	74.67	76.60	73.05
ResNet-50 [38]	80.57	78.02	79.21	76.95
NASNet [45,70]	79.23	76.28	77.25	75.44
DenseNet-201 [41]	84.75	83.76	85.21	82.42
Proposed	**85.92**	**84.69**	**85.33**	**84.11**

**Table 14:** The *t*-test analysis results between our model and the second-best and third-best models.

Comparisons		*p*-Value	Confidence Level
Proposed	Second-best	0.03	97%
Proposed	Third-best	7.84 × 10^−9^	99%

**Table 15:** Summary of two-fold cross-validation of training and testing data for open-world configuration (unit: images).

Validation	Training			Testing		
	Classes	Original	Augmented	Classes	Original	Total
1st fold-A	Cofield, Depuy	377	13,572	Tornier, Zimmer	220	14,169
1st fold-B	Tornier, Zimmer	220	7920	Cofield, Depuy	377	8517
2nd fold-A	Tornier, Cofield	154	5544	Zimmer, Depuy	443	3585
2nd fold-B	Zimmer, Depuy	443	15,948	Tornier, Cofield	154	16,545

**Table 16:** Comparison of our proposed model with the second- and third-best models of Table 13 for open-world configuration (unit: %).

CNN Model	Accuracy	F1-Score	Precision	Recall
ResNet-50 [38]	74.96	67.14	67.78	66.51
DenseNet-201 [41]	76.64	71.31	70.64	72.05
Proposed	77.36	70.85	71.22	70.49

## Data Availability

Not applicable.
